# Feasibility and Acceptability of Ecological Momentary Assessment With Young Adults Who Are Currently or Were Formerly Homeless: Mixed Methods Study

**DOI:** 10.2196/33387

**Published:** 2022-03-25

**Authors:** Sara Semborski, Benjamin Henwood, Brian Redline, Eldin Dzubur, Tyler Mason, Stephen Intille

**Affiliations:** 1 Suzanne Dworak-Peck School of Social Work University of Southern California Los Angeles, CA United States; 2 School of Medicine Oregon Health and Science University Portland, OR United States; 3 Department of Population and Public Health Science Keck School of Medicine University of Southern California Los Angeles, CA United States; 4 Khoury College of Computer Sciences Northeastern University Boston, MA United States; 5 Bouvé College of Health Sciences Northeastern University Boston, MA United States

**Keywords:** ecological momentary assessment, homelessness, young adults, reactivity, compliance, mobile phone

## Abstract

**Background:**

Ecological momentary assessment (EMA) has been used with young people experiencing homelessness to gather information on contexts associated with homelessness and risk behavior in real time and has proven feasible in this population. However, the extent to which EMA may affect the attitudes or behaviors of young adults who are currently or were formerly homeless and are residing in supportive housing has not been well investigated.

**Objective:**

This study aims to describe the feedback regarding EMA study participation from young adults who are currently or were formerly homeless and examine the reactivity to EMA participation and compliance.

**Methods:**

This mixed methods study used cross-sectional data collected before and after EMA, intensive longitudinal data from a 7-day EMA prompting period, and focus groups of young adults who are currently or were formerly homeless in Los Angeles, California, between 2017 and 2019.

**Results:**

Qualitative data confirmed the quantitative findings. Differences in the experience of EMA between young adults who are currently or were formerly homeless were found to be related to stress or anxiety, interference with daily life, difficulty charging, behavior change, and honesty in responses. Anxiety and depression symptomatology decreased from before to after EMA; however, compliance was not significantly associated with this decrease.

**Conclusions:**

The results point to special considerations when administering EMA to young adults who are currently or were formerly homeless. EMA appears to be slightly more burdensome for young adults who are currently homeless than for those residing in supportive housing, which are nuances to consider in the study design. The lack of a relationship between study compliance and symptomatology suggests low levels of reactivity.

## Introduction

### Background

Ecological momentary assessment (EMA), also known as the *experience sampling method*, is an intensive, longitudinal, real-time sampling strategy with widespread adoption in public health research and social sciences [1-4]. EMA, which leverages advancements in mobile computing to deliver repeated surveys throughout a specified measurement period, often by using cell phone technology [5], can reduce recall biases and improve the ecological validity and environmental representativeness of the collected data [6]. Given that young adults experiencing homelessness, of which the prevalence is estimated to be 1 in 10 annually in the United States [7], live relatively unstable lives that are highly affected by their immediate environment [8,9] and have high rates of mobile phone technology adoption [10], EMA may be well suited for use with young adults experiencing homelessness [11,12]. In addition, this method may overcome existing limitations in homelessness research that has relied on methods using retrospective reporting, including cross-sectional designs (eg, single point-in-time measurement) [13] and longitudinal study designs with infrequent measurement points (eg, monthly follow-ups) that include issues with attrition [14]. However, although EMA is likely to be a useful method in homelessness research, it is important to better understand the feasibility and acceptability of EMA in this population.

To date, few studies have used EMA to investigate the daily life experiences of young adults who have experienced homelessness, and several studies have shown the feasibility of EMA in this population. Santa Maria et al [15] provided smartphones to 66 young adults who were homeless aged 18 to 25 years to collect EMAs over 21 days and found daily drug use to be predicted by discrimination, pornography use, alcohol use, and urges for substance use and stealing behaviors. In a different study, Tyler et al [16] distributed mobile phones to implement EMA via SMS text messaging with 150 youths who were homeless aged 16 to 22 years over a 30-day period and found that experiencing physical or sexual victimization on a specific day was positively associated with drinking alcohol later that day. Both studies reported high compliance with completing EMA prompts, and the latter study also reported that participants perceived the study to be of low burden [17].

Another important aspect of EMA feasibility is reactivity. Reactivity is understood to be the extent to which the frequency or quality of behavior changes as a result of being monitored [18]. Understanding the potential reactivity is critically important as it suggests that EMA could serve as a possible intervention or manipulation. The literature has generally found low reactivity when using EMA [19-23], including in college and clinical samples [24,25]. Acorda et al [26] found that young people experiencing homelessness were highly receptive to EMA but may have experienced limitations regarding the use of technology and that the repetition of EMA prompts may have affected some behaviors of those participating. However, the extent to which EMA may affect the attitudes or behaviors of young adults experiencing homelessness during a time of identity formation and instability, especially when examining risk behaviors, including sex risk and substance use, has not been well investigated.

### Objective

More research is needed to understand the daily experiences of young people who have experienced homelessness, including those who have transitioned into supportive housing, which is a primary intervention being applied to homelessness. Previous work has identified ways in which housed and unhoused young adults differ, including abuse at home [27], which affects mental health [28,29] and substance use [30]. To understand the environmental influences on young adults who have experienced homelessness, it is imperative to examine both those who are currently experiencing homelessness and those who have transitioned from homelessness to supportive housing environments. This study seeks to address this gap by using a mixed methods approach to examine whether there are differences between young adults who are currently (ie, unhoused) versus were formerly homeless (ie, housed in supportive housing) in terms of acceptability, compliance, and reactivity to EMA.

## Methods

### Study Design

This mixed methods study examines the experiences of EMA in a sample of young adults currently experiencing homelessness and young adults who were formerly homeless who have been placed into supportive housing programs. Specifically, as described in a previously published research protocol paper, young adults participated in a study on health risk behaviors using geographic EMA through a smartphone app that allowed for the collection of time-stamped geographic location data along with EMA behavioral data. Consistent with previous literature [15-17], high compliance with completing EMA prompts (80.2% across the entire sample) over a 1-week study period for the combined sample has already been reported [31]. For this study, we first compared the feedback of housed and unhoused participants regarding their experiences of participating in the EMA week. Responses were then used to examine rates and predictors of EMA compliance and survey responses regarding acceptability and feasibility, comparing those in housing with those who were currently homeless (ie, unhoused). Reactivity was examined using reported anxiety and depression symptomatology before and after the EMA week. Next, we used a qualitative approach to analyze the focus group data of participants who are current or were formerly homeless to better understand their experiences with EMA, which may help explain our quantitative findings.

### Participants

Participants (N=231) in transitional living programs or permanent supportive housing (ie, *housed* sample; n=122, 52.8%) and participants not in housing programs (ie, *unhoused* sample; n=109, 47.2%) were enrolled in the study in Greater Los Angeles using stratified convenience sampling. Unhoused participants were recruited via drop-in centers and emergency shelters, including individuals who were explicitly homeless or unstably housed with temporary living situations that were not reliable beyond 30 days (eg, temporarily crashing with a friend or family member or couch surfing). Participants who consented to the EMA component of the study received up to US $90 in scaled compensation based on response rates and were given the choice of using a study phone with an unlimited data plan or their own smartphone, with an additional US $10 compensation for using their own data plan.

### Ethics Approval

All protocols and procedures were approved by the institutional review board at the University of Southern California (review number: UP-16-00046) [31].

### Quantitative Component

#### Overview

Participants were enrolled in a 7-day EMA study comprising questions that asked participants to report on current and previous 2-hour experiences, which were delivered approximately every 2 hours during waking hours using a custom-built app for smartphones using the Android operating system (Google). This study used custom EMA software written by the investigative team. Phones were programmed to only deliver prompts during the waking day, which was determined using the participants’ individual estimated sleep and wake times. Prompted surveys asked about physical and social environments, as well as affect and substance use. Participants received an average of 5 EMA prompts per day.

In addition to the EMA prompts, participants completed a daily survey for each EMA day. Daily diaries captured the risk behaviors of the previous day and infrequent behaviors that may be missed by EMAs. Daily survey prompts were scheduled to be delivered at a participant’s preferred time but were also available to access via the app at any time during the day to report on the previous day. Daily surveys inquired about participants’ social environments, sex behaviors, and substance use.

Before the EMA week, participants completed a baseline interview that took an average of 60 minutes to gather demographic information and data on their histories of homelessness, mental health, and other behaviors. Following the EMA week, participants participated in an exit survey, in which their thoughts and feelings regarding their participation in the EMA study were gathered. The exit surveys lasted approximately 30 minutes. Participants returned the phones and were paid for study participation at the conclusion of the exit survey. The Patient Health Questionnaire-9 was used to assess depression symptomatology [32], and the General Anxiety Disorder-7 was used to assess anxiety symptomatology [33] at both the baseline and exit surveys.

To reduce missed surveys, there were multiple push notifications for both the EMA and daily survey prompts. EMA prompts required a response within 10 minutes after the first prompt, which comprised a chime and vibration. During this 10-minute window, push notifications were sent every 3 minutes. After 10 minutes, the EMA prompt became inaccessible to ensure momentary reporting of the current time and day. Daily surveys were programmed to send push notifications at 3 time points during the day; however, they could be answered at any point within the day; when answering the daily surveys, participants reported on the prior waking day. The complete study methods are available for further review elsewhere [31] (see Multimedia Appendix 1 for the complete EMA questionnaire and Multimedia Appendix 2 for the daily survey questions).

#### Analyses

Quantitative analyses in this study included chi-square analysis to compare results by housing status and bivariate linear regressions to predict EMA and daily compliance. Compliance measures the total number of prompts answered out of those received. As the aim of this study was to examine personal factors, such as housing status, rather than artifacts associated with EMA technology, which are associated with compliance, we chose to calculate compliance based on the number of prompts received instead of prompts possible (ie, scheduled). Furthermore, between-subject mixed effects regressions assessed reactivity using the Patient Health Questionnaire-9 [32] and General Anxiety Disorder-7 [33], with random intercepts for each participant.

### Qualitative Component

#### Overview

A total of 4 separate focus groups were conducted to better understand participants’ experiences with EMA, each of which occurred within 2 weeks of their last day in the study and lasted approximately 1 hour. Focus groups were chosen as a time-saving way of easily measuring and capturing wide-ranging reactions to EMA. A total of two focus groups included participants recruited from housing programs (one with n=12 transitional living program residents and one with n=6 permanent supportive housing program residents) and 2 focus groups (n=6 and n=7) recruited from youth drop-in centers. Focus group facilitators began by asking participants about their general experiences in the study (eg, “What did you like? What did you not like?”*)*. Additional probing questions included specific aspects of study participation (eg, whether EMA interfered with their daily lives), perceived reactivity to EMA surveys, how accurate they thought their reporting was, level of comfort reporting about sensitive topics such as drugs and alcohol, timing and density of survey prompts, and suggestions for similar studies in the future.

#### Analyses

Focus group recordings were transcribed and evaluated by 2 independent reviewers, one of whom was the focus group facilitator. Coding and case summaries took a deductive approach using exit survey questions focused on experiences of study participation as a guide (see Multimedia Appendix 3 for the focus group interview guide). Co-coder consensus was achieved through the codevelopment of 4 individual case summaries that summarized each item, including quotations, with 1 for each focus group. Case summaries were then analyzed, first considering housed and unhoused groups separately and then together, to see what might account for and expand upon the differences found.

## Results

### Quantitative: Exit Interview by Housing Status

Table 1 describes the sample characteristics by housing status, and Table 2 describes the exit surveys and responses, also by housing status. Approximately two-thirds of the participants had an overall positive experience with the study, >90% reported they would be willing to participate in the study again, and approximately 67.5% (156/231) reported that the study took place during a typical week. Approximately 69.7% (161/231) did not feel judged about their sex or drug use, and approximately half of the participants would prefer to use their own phone in a new study provided they owned a phone compatible with EMA technology. As a result of personal choice and incompatibility, only 9% (21/231) used a personal phone in this study, and significantly more unhoused individuals opted for personal phone use. Housed and unhoused participants also reported statistically significant differences in their experiences of charging the phones, behavior change because of EMA content, being open and honest about EMA survey questions, and whether EMA interfered with their daily life. Compared with those in housing, unhoused participants reported greater difficulty charging their phones (*P*=.007 to overall *P*=.02), greater self-perceived behavior changes in response to EMA (*P*=.001 to overall *P*<.001), that EMA interfered more with their daily life (specific and overall *P*<.001), and more stress or anxiety because of EMA surveys (*P*=.008 to overall *P*=.02), whereas housed participants reported that they were more comfortable answering EMA survey questions openly and honestly (overall *P*=.03).

**Table 1:** Sample characteristics by housing status (N=231).

Characteristics		Housed (n=122)	Unhoused (n=109)	All participants	*P* value	
Age (years), mean (SD)		22.6 (2.4)	21.8 (2.0)	22.2 (2.3)	.007	
**Gender, n (%)**						.06
	Male	56 (45.9)	67 (61.5)	123 (53.3)		
	Female	46 (37.7)	31 (28.4)	77 (33.3)		
	Gender nonconforming, expansive, or transgender	20 (16.4)	11 (10.1)	31 (13.4)		
Sexual minority, n (%)		58 (47.5)	51 (46.8)	109 (47.2)	.91	
**Race or ethnicity, n (%)**						.01
	White	11 (9)	10 (9.2)	21 (9.1)		
	Black	31 (25.4)	51 (46.8)	82 (35.5)		
	Hispanic or Latino	31 (25.4)	17 (15.6)	48 (20.8)		
	Biracial or multiracial	36 (29.5)	25 (22.9)	61 (26.4)		
**Lifetime homelessness (years), n (%)**						.86
	<1	45 (36.9)	46 (42.2)	91 (39.4)		
	1-2	32 (26.2)	25 (22.9)	57 (24.7)		
	3-4	23 (18.9)	19 (17.4)	42 (18.2)		
	≥5	22 (18)	19 (17.4)	41 (17.6)		
EMA^a^ compliance, mean (SD)		0.80 (0.15)	0.78 (0.19)	0.79 (0.17)	.39	
Daily compliance, mean (SD)		0.914 (0.15)	0.906 (0.17)	0.910 (0.16)	.71	

^a^EMA: ecological momentary assessment.

**Table 2:** Exit survey by housing status (N=231).

Survey questions		Housed (n=122)	Unhoused (n=109)	All participants	*P* value	
**General experience, n (%)**						.66
	Very negative or somewhat negative	0 (0)	1 (0.9)	1 (0.4)		
	Neutral	27 (22.1)	33 (30.3)	60 (25.9)		
	Very positive or somewhat positive	55 (45)	65 (59.6)	120 (51.9)		
**Difficulty charging, n (%)**						.02
	Not at all or a little bit	62 (50.8)	61 (55.9)	123 (53.2)		
	Somewhat^a^	5 (4)	15 (14.1)	20 (8.7)		
	Quite a bit or very much^a^	10 (8.1)	23 (21.1)	33 (14.3)		
**Behavioral change, n (%)**						<.001
	Disagree or strongly disagree^a^	74 (60.7)	36 (33)	110 (47.6)		
	Neither agree nor disagree^a^	17 (13.9)	27 (24.8)	44 (19)		
	Agree or strongly agree	17 (13.9)	36 (33)	53 (22.9)		
**Openness or honesty, n (%)**						.03
	Disagree or strongly disagree	4 (3.3)	2 (1.8)	6 (2.6)		
	Neither agree nor disagree	2 (1.6)	10 (9.1)	12 (5.2)		
	Agree or strongly agree	102 (83.6)	87 (79.8)	189 (81.8)		
**Interference with life, n (%)**						<.001
	Not at all or a little bit	75 (61.5)	45 (41.3)	120 (51.9)		
	Somewhat^a^	23 (18.9)	22 (20.2)	45 (19.5)		
	Quite a bit or very much^a^	9 (7.4)	32 (29.4)	41 (17.7)		
**Stress or anxiety, n (%)**						.02
	Not at all or a little bit	96 (78.7)	74 (67.9)	170 (73.6)		
	Somewhat^a^	8 (6.6)	12 (11)	20 (8.7)		
	Quite a bit or very much^a^	4 (3.3)	13 (11.9)	17 (7.4)		
**Willingness to follow up, n (%)**						.07
	No	2 (1.6)	7 (6.4)	9 (3.9)		
	Yes	106 (86.9)	92 (84.4)	198 (85.7)		
**Feeling judged, n (%)**						.99
	Disagree or strongly disagree	84 (68.9)	77 (70.6)	161 (69.7)		
	Neither agree nor disagree	10 (8.1)	9 (8.2)	19 (8.2)		
	Agree or strongly agree	14 (11.5)	13 (11.9)	27 (11.7)		
**Willingness to participate again, n (%)**						.48
	Neither agree nor disagree	7 (5.7)	9 (8.2)	16 (6.9)		
	Agree or strongly agree	100 (81.9)	89 (81.7)	189 (81.8)		
**Phone preference (new study), n (%)**						.80
	Study phone	57 (46.7)	54 (49.5)	111 (48.1)		
	Personal phone	51 (41.8)	45 (41.3)	96 (41.6)		
**Phone used this study, n (%)**						.07
	Personal phone	7 (5.7)	14 (12.8)	21 (90.9)		
	Study phone	101 (82.8)	85 (77.9)	186 (80.2)		
**Typical week, n (%)**						.14
	No, this week was not typical	22 (18)	29 (26.6)	51 (41.8)		
	Yes, this was a typical week	86 (70.5)	70 (64.2)	156 (67.5)		

^a^Combined for specific *P* values.

### Quantitative: Delivery and Compliance

**Figure 1:**
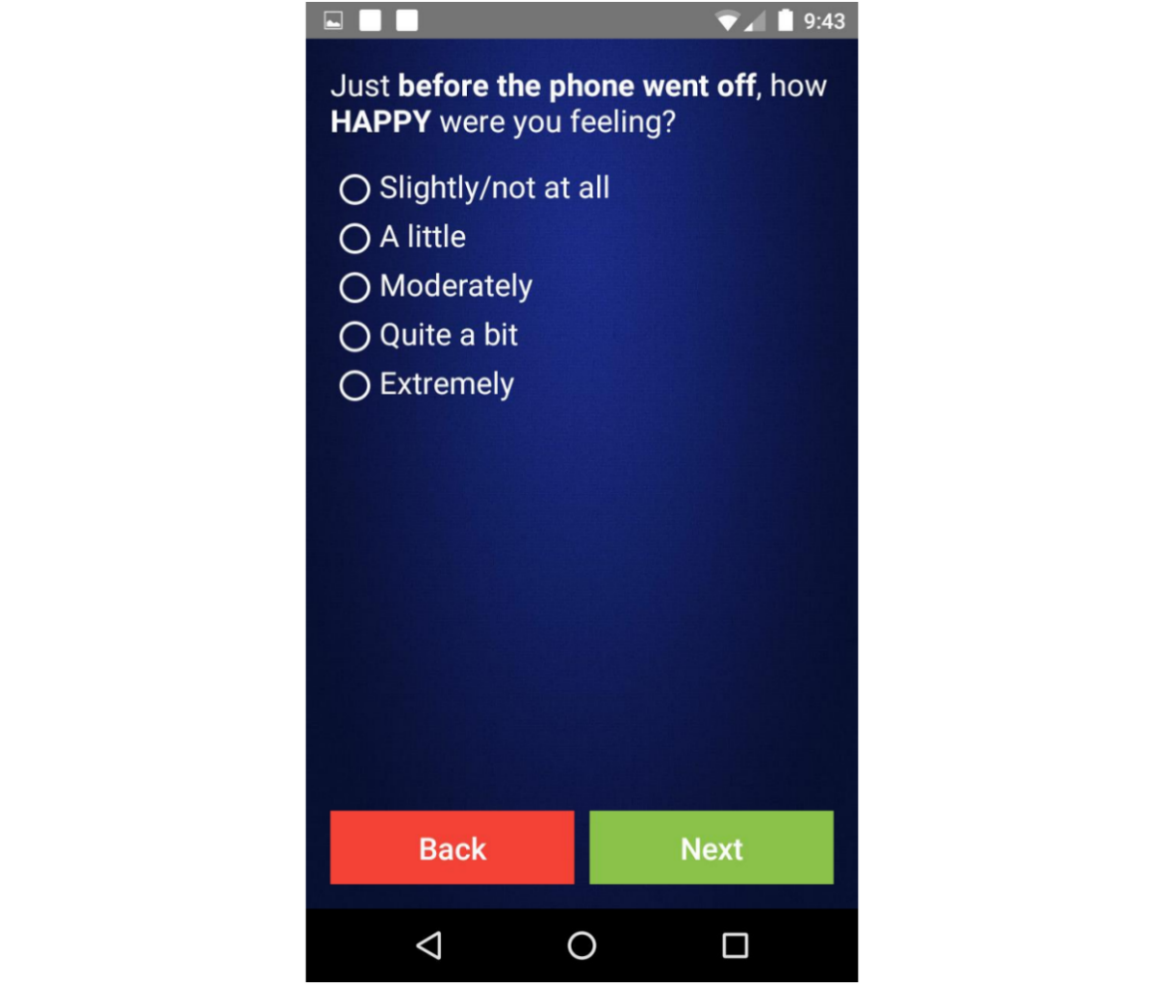
Screenshot of the ecological momentary assessment presented to participants.

Out of a theoretical maximum of 20,076 prompts, 17,944 (89.38%) prompts were scheduled for delivery by the custom software. The discrepancy of the 10.62% (2132/20,076) of prompts may be explained by hardware issues (eg, low battery), software issues (eg, app crashing), or schedule timing (ie, where a participant was enrolled at midday and would not have received earlier prompts). Of the scheduled prompts, approximately 39.57% (7101/17,944) were not delivered as the app detected that the prompt time was within sleep parameters specified by the participant. Of the 17,944 prompts, 679 (3.78%) scheduled prompts were not delivered because of Android system features designed to conserve battery life and memory, and 138 (0.77%) prompts were not delivered as the phone was intentionally turned off. The remaining undelivered scheduled prompts, which was an average of 46, were missing because of unknown software or hardware errors. In all, of the 17,944 prompts, the participants received 9980 (55.62%) prompts and completed 8001 surveys upon answering the prompts (8001/9980, 80.17% EMA completion). Participants failed to answer 18.24% (1820/9980) of prompts and answered but did not complete 1.59% (159/9980) of surveys. Participants took, on average, 97 (SD 70, range 19-599) seconds to complete the EMA surveys and completed 13.8 (SD 6.4, range 0-25) questions per EMA survey (see Multimedia Appendix 1 for the EMA survey questions and Figure 1 for an example screenshot of the app).

Approximately 3% (7/231) of participants did not receive 2.93% (49/1673) of daily survey prompts, and an additional 6.69% (112/1673) of daily survey prompts were not delivered, out of the theoretical maximum number of prompts, because of possible hardware, software, or schedule timing issues. Of the 1512 scheduled daily survey prompts, 5 (0.33%) were not delivered because of Android system battery conservation issues, and 5 (0.33%) were not delivered as the phone was turned off by the participant. Out of 1502 prompts that were received, participants answered and completed 1376 (91.61% daily compliance) daily prompts; of the 1512 surveys, participants failed to fully complete 6 (0.4%) surveys. Participants took, on average, 89 (SD 61, range 14-570) seconds to complete daily surveys, and they completed 12.2 (SD 5.1, range 0-45) questions per daily survey (see Multimedia Appendix 2 for the daily survey questions).

The results of the analyses of compliance are presented in Table 3. Neither daily compliance nor EMA compliance was associated with housing status (*t*_1_=−0.38, *P*=.71 and *t*_1_=−0.86, *P*=.39, respectively). Only 9.1% (21/231) of participants chose to complete the study on their own phone, with no difference in compliance between those who used a personal versus a study phone (*t*_1_=1.15, *P*=.25 and *t*_1_=0.74, *P*=.46, respectively). Compared with those reporting negative or neutral experiences, participants who reported a *very positive* or *somewhat positive* experience with the study had 6% (SE 0.03%) greater EMA compliance (*t*_1_=2.49; *P*=.01). Furthermore, those reporting *not at all* or *a little bit* of difficulty charging their device had 6% (SE 0.03%) greater EMA compliance than those reporting more difficulty charging their device (*t*_1_=2.41, *P*=.02).

Daily survey compliance was not associated with general experience with the study (*t*_1_=0.97; *P*=.33), nor was participants’ self-report of honesty regarding survey responses associated with EMA or daily compliance (*t*_1_=0.92, *P*=.36 and *t*_1_=1.23, *P*=.22, respectively). Compared with those reporting somewhat or greater interference with the study protocol in their lives, participants who reported not at all to a little bit of interference had 8% (SE 0.02%) greater EMA compliance (*t*_1_=−3.83; *P*<.001) and 6% (SE 0.02%) greater daily compliance (*t*_1_=−3.42; *P*=.001). Similarly, participants experiencing little or no stress or anxiety from surveys had 7% (SE 0.03%) greater EMA compliance (*t*_1_=2.62; *P*=.009) and 5% (SE 0.03%) daily compliance (*t*_1_=2.05; *P*=.04) than those experiencing greater stress or anxiety from surveys. Compared with those who indicated feeling judged by surveys, participants who did not endorse feeling judged by the surveys had 6% (SE 0.03%) greater EMA compliance (*t*_1_=2.38; *P*=.02) and 9% (SE 0.02%) greater daily compliance (*t*_1_=4.02; *P*<.001). Participants who reported having a typical week had 8% (SE 0.02%) greater EMA compliance than those who reported having an atypical week (*t*_1_=3.58; *P*<.001); however, those with typical weeks only had marginally greater daily compliance (*t*_1_=1.79; *P*=.08). Willingness to participate again was not associated with EMA or daily compliance (*t*_1_=0.34, *P*=.74 and *t*_1_=−0.05, *P*=.96, respectively).

**Table 3:** Bivariate linear regressions of compliance (N=231).

Characteristics		Sample size, n (%)	EMA^a^ compliance				Daily compliance		
			Margin (SE)	β^b^ (SE; 95% CI)	*P* value	Margin (SE)		β^b^ (SE; 95% CI)	*P* value
**Housing status**									
	Unhoused (reference)	231 (100)	0.78 (0.02)	N/A^c^	N/A	0.91 (0.02)		N/A	N/A
	Housed	231 (100)	0.80 (0.02)	.02 (.02; −0.03 to 0.64)	.39	0.91 (0.01)		.008 (.02; −0.03 to 0.05)	.71
**Phone type**									
	Personal phone (reference)	207 (89.6)	0.78 (0.03)	N/A	N/A	0.89 (0.03)		N/A	N/A
	Study phone	207 (89.6)	0.81 (0.01)	.03 (.04; −0.05 to 0.10)	.46	0.93 (0.01)		.04 (.03; −0.03 to 0.10)	.25
**Study experience**									
	Negative or neutral (reference)	181 (78.4)	0.76 (0.02)	N/A	N/A	0.93 (0.01)		N/A	N/A
	Positive	181 (78.4)	0.82 (0.01)	.06 (.03; 0.01 to 0.11)	.01	0.91 (0.02)		.02 (.02; −0.02 to 0.06)	.33
**Difficulty charging**									
	None or a little (reference)	176 (76.2)	0.83 (0.01)	N/A	N/A	0.94 (0.01)		N/A	N/A
	Somewhat or yes	176 (76.2)	0.76 (0.02)	−.06 (.03; −0.11 to −0.01)	.02	0.90 (0.02)		−.04 (.02; −0.08 to 0.004)	.07
**Behavior change**									
	Yes or neutral (reference)	207 (89.6)	0.79 (0.02)	N/A	N/A	0.92 (0.01)		N/A	N/A
	No	207 (89.6)	0.82 (0.02)	.04 (.02; −0.004 to 0.08)	.08	0.92 (0.01)		−.0008 (.02; −0.04 to 0.04)	.97
**Openness or honesty**									
	No or neutral (reference)	207 (89.6)	0.77 (0.04)	N/A	N/A	0.88 (0.03)		N/A	N/A
	Yes	207 (89.6)	0.80 (0.01)	.04 (.04; −0.04 to 0.11)	.36	0.93 (0.01)		.04 (.03; −0.03 to 0.11)	.22
**Interference with life**									
	None or a little (reference)	206 (89.2)	0.84 (0.01)	N/A	N/A	0.95 (0.01)		N/A	N/A
	Somewhat or yes	206 (89.2)	0.76 (0.02)	−.08 (.02; −0.13 to −0.04)	<.001	0.89 (0.01)		−.07 (.02; −0.10 to −0.03)	.001
**Stress or anxiety**									
	None or a little (reference)	207 (89.6)	0.82 (0.01)	N/A	N/A	0.93 (0.01)		N/A	N/A
	Somewhat or yes	207 (89.6)	0.74 (0.03)	−.07 (.03; −0.13 to −0.02)	.009	0.88 (0.02)		−.05 (.03; −0.10 to −0.002)	.04
**Feeling judged**									
	Yes or neutral (reference)	207 (89.6)	0.76 (0.02)	N/A	N/A	0.85 (0.02)		N/A	N/A
	No	207 (89.6)	0.82 (0.01)	.06 (.03; 0.01 to 0.11)	.02	0.94 (0.01)		.09 (.02; 0.05 to 0.14)	<.001
**Typical week**									
	No (reference)	207 (89.6)	0.74 (0.02)	N/A	N/A	0.89 (0.02)		N/A	N/A
	Yes	207 (89.6)	0.83 (0.01)	.09 (.02; 0.04 to 0.14)	<.001	0.93 (0.01)		.04 (.02; −0.004 to 0.08)	.08
**Willingness to participate again**									
	Neither agree nor disagree (reference)	205 (88.7)	0.79 (0.04)	N/A	N/A	0.93 (0.04)		N/A	N/A
	Yes, agree	205 (88.7)	0.81 (0.01)	.01 (.04; −0.07 to 0.10)	.74	0.92 (0.01)		−.002 (.04; −0.07 to 0.07)	.96

^a^EMA: ecological momentary assessment.

^b^Indicates percentage change in compliance.

^c^N/A: not applicable.

### Quantitative: Reactivity Analyses

Table 4 displays the results from the mixed effects models to examine reactivity to EMA participation, specifically regarding anxiety and depression symptomatology. Both anxiety and depression scores decreased from baseline to follow-up (β=−1.77, *P*<.001 and β=−1.10, *P*=.03, respectively). However, no significant effects for EMA compliance were detected for either anxiety or depression; thus, the decrease in symptomatology was not associated with compliance. Both models controlled for age, gender, sexual orientation, race and ethnicity, and housing status.

**Table 4:** Mixed effects regressions for reactivity (N=231).

Characteristics		Anxiety (GAD-7^a^)		Depression (PHQ-9^b^)	
		β (95% CI)	*P* value	β (95% CI)	*P* value
Time point (*j*)		−1.79 (−2.62 to −0.96)	<.001	−1.08 (−2.07 to −0.09)	.03
Compliance (EMA^c^)		1.11 (−2.88 to 5.10)	.59	.62 (−3.62 to 4.86)	.78
Housing status (housed)		−.02 (−1.49 to 1.45)	.98	−.58 (−2.14 to 0.98)	.47
Age (years)		.14 (−0.17 to 0.46)	.37	.01 (−0.32 to 0.35)	.94
**Gender (reference: male)**					
	Female	.21 (−1.34 to 1.77)	.79	−.12 (−1.77 to 1.54)	.89
	Gender nonconforming, expansive, or transgender	2.24 (−0.11 to 4.59)	.06	3.77 (1.28 to 6.25)	.003
Sexual minority (reference: heterosexual)		3.63 (2.17 to 5.09)	<.001	3.64 (2.10 to 5.19)	<.001
Race (Black)		−.99 (−2.59 to 0.60)	.22	−1.27 (−2.96 to 0.42)	.14
Hispanic		1.11 (−0.48 to 2.71)	.17	1.02 (−0.67 to 2.71)	.24

^a^GAD-7: General Anxiety Disorder-7.

^b^PHQ-9: Patient Health Questionnaire-9.

^c^EMA: ecological momentary assessment.

### Qualitative Findings

#### Overview

The qualitative findings that were generated independently of the quantitative findings were categorized under the 5 main emergent themes. The first theme explains how participants felt the study design *increased mindfulness and reflection*, whereas the second theme captures those negative instances when the participation in the study resulted in *causing stress and anxiety*. The third theme discusses the ways in which study participation incited behavior change, whereas the fourth theme addresses participants *responding honestly* to questions. The final theme captures participant suggestions about future study designs. We note that a comparative analysis of the housed and unhoused samples indicated that these themes apply to both groups, as shown in Table 5.

**Table 5:** Qualitative findings by housing status.

Themes	Housed focus groups (n=18)	Unhoused focus groups (n=13)
Increased mindfulness and reflection	“You get to know yourself. Like how many times do I do these things a day? Who am I around every day? What am I doing every day? So, it gave me insight on who you are and what you do every day. Because sometimes we'll just do things. And we don’t keep track of those things. They kind of keep you on track a little bit.” [SP^a^102] “Some stuff I forgot about, that...It just opened my mind more. I was, ‘Okay, I need to start paying more attention to that. I need to start paying more attention to this. I am around certain people who do stuff like this.’” [SP209]	“It was the shit to me. It actually calmed me down on most occasions.” [SP305] “It makes you feel good. You want to get up and answer the questions that, how you feel, how you feel going out today, and shit like that. It made me feel good, honestly.” [SP306] “Yeah ‘cause I forgot to do my chores and as soon as that survey come on, and be like, ‘Oh snap. I forgot to do my chore.’...But then I’m right back on it.” [SP403] “It also gave me a good understanding of how when I hang out with these certain people, yeah, I am smoking more. And if I hang out with this certain people, I am drinking more...Gonna be like, okay dude, I’m hanging out with you but just because we’re hanging out doesn’t mean we have to drink. You know? If they’re drinking it’s their choice. Alright man, I’m noticing I can’t be hanging out with you every day. You’re drinking every day, I’m hanging out with you every day, I’m probably gonna be drinking every day.” [SP406]
Causing stress and anxiety	“Well, it was irritating, like once or twice. I thought it was going to ask different questions. I didn’t know about the repetition thing.” [SP106] “Sometimes it would annoy me, like, ‘How do you feel?’ Like, ‘Oh, I feel annoyed now.’” [SP207]	“I got paranoid sometimes, like if I was hanging out with these people and it was asking me, ‘who are you with?’ ‘Did you do any drugs with them?’...It was just about the whole street thing; it feels like snitching.” [SP301] “I got annoyed sometimes. If I was going through something and the survey went off, I just didn’t want to answer it sometimes.” [SP302] “I did [feel uncomfortable] at first. I thought it was like the Feds or something. I was like oh shit, I’m not gonna lie, I was doing all types of lies though. A few of the surveys I feel like I failed them or something. I don’t know. That’s just how my mentality think. I felt like the phone was recording. Something. On everything. Yeah, I felt like the Feds was watching. And taking video at the same time. I let the phone die for like a day and a half...I’m like I don’t know, I’m gonna keep the phone off. I was just annoyed about it because like they know oh really alerted when I got a text that ‘we see your phone hasn’t been charged.’ [laughs] You guys are watching me! I know what you can do with technology. It’s a simple program, there’s no telling what’s written in that program that I don’t see.” [SP403]
Inciting behavior change	“I’m not going to drink today, because they’re going to ask me how many drinks I drank.” [SP102] “I had kind of like a Pavlov’s dog affect, where every time it went off, I’m like, ‘Ooh, I could really use some alcohol right now.’ I’m like, ‘Ooh, this is reminding me that alcohol is not a great option, but it is an option.’ [SP206]	“Have you smoked yet today? Okay? I haven’t, mostly because I haven’t got my weed so hold on, thanks for letting me know I have to go get weed. That’s what I’m saying. Reminding me to go get my marijuana when I run out.” [SP305] “On the alcohol and drink question I went like...the repetitive asking you another question and just mentioning a drink, made me want to drink. I never had that many drinks in a week.” [SP403]
Responding honestly	“I tried to be as honest as possible. Because I knew that it was a study. So I tried to be as honest...Because you guys are going to look at it and try to get real answers from people. It was hard. But I tried to be honest about it.” [SP102] “The thing about surveys too, is it’s a non-judgemental environment that you’re telling your information to.” [SP105] “Yeah, I feel like if anything it makes me feel more comfortable, because the phone, you don’t have to give a fuck...I’m going to be real today, and I’m going to be real to myself. It’s kind of like a diary if you think about it. That survey was like a diary for a week...If you have time for that.” [SP205] “To be honest, I lied on a few questions...I lied because- It just reminded me of what a whore I am, because like...so it would be like ‘How many times did you have sex today?’ And stuff like that, I’m like ‘Ooh.’ [SP202]	“...it was just about the whole street thing, it feels like snitching. Yep.” [SP301] “I say it depends on the mood of how people will feel when I’m doing the surveys.” [SP306] “A lot of them I tried to be honest some, some of them I kind of like, I don’t think I put the right answers, like it would say, where you at, and sometimes I’d put like, or what are you doing, and I’d say hanging out, but really I was eating, still hanging out a little bit, so kind of lie about that, I need to be more honest, cause you know, I don’t want them to know exactly what I’m doing.” [SP404] “I thought it was like the Feds or something. I was like oh shit, I’m not gonna lie, I was doing all types of lies though.” [SP403]
Suggestions for future studies	“I think [using my own phone] was more convenient. It was a lot easier than having to have a second phone. Losing track of it.” [SP105] “I thought it was interesting. It was cool. I think I prefer it to be on my actual phone than another phone. Because it was kind of hard to keep up with it.” [SP104] “It felt extra for me but that’s because...I’m really bad about keeping my regular phone on me, so I said I was a bad millennial. I’m a bad millennial.” [SP204] “It’s hard to remember to carry two phones.” [SP208] “But even if I went from my bedroom to the dining room, and thirty minutes went by and I was like, ‘I have to go get my phone.’ Then I saw that I missed one survey.” [SP204] “I feel like I have bigger problems than carrying two phones. It wasn’t the hardest thing in my life, but sometimes I would forget it and be like, ‘Aw, crap.’ That’s it.” [SP207] “It was hard for me. I was literally driving. I always keep my phone away. I’m over there trying to jump in the purse like: Where is this phone? And then, another thing, my neighbor...My neighbor [who was also in the study] always comes over...And my other neighbor...So he’d be like: ‘Who’s phone is who’s? Who got that...’ And I’d be like: ‘Do you have my phone? That’s my phone?’ How did we know it was our phone?...It took me about a day, or maybe two, to actually [get used to it].” [SP104]	“[Borrowing] the phone...actually helped me with my daily life, bro. Because I didn’t have a phone at the time, so it helped me to get phone calls.” [SP404] “Oh yeah, me too. Google maps.” [SP408] “I hadn’t had a phone since that one. I do want a phone for another week though.” [SP404] “Because it would ask have you did this in the past 2 hours and I might forget. Or how many cigarettes, how many times do you think you used tobacco products. Like, I don’t know, let me count the cigarettes in my pack right now...I might forget and I just smoked a cigarette 5 minutes before it happened. So more every time you smoke a cigarette, log it in the phone. Tally it.” [SP402]

^a^SP: study participant

#### Increased Self-reflection and Self-awareness

Self-reflection and awareness while participating in EMA were common points of discussion in the focus groups. In fact, this idea came up in all 4 focus groups. Many participants noted increased self-awareness regarding how their own thoughts, feelings, and social or physical contexts influenced their engagement in protective or risky behaviors.

For example, several participants noted that the EMA helped them stay on track over the course of the day, including triggering better awareness of time, what has been accomplished, and what has yet to be done. A participant discussed being able to keep track of what they were up to:

You get to know yourself. Like how many times do I do these things a day? Who am I around every day? What am I doing every day? So, it gave me insight on who you are and what you do every day. Because sometimes we'll just do things. And we don’t keep track of those things. They kind of keep you on track a little bit.

Study participant (SP) 102, housed

Similarly, another participant specifically talked about how EMA kept them productive and on top of their chores, saying the following:

Yeah ‘cause I forgot to do my chores and as soon as that survey come on, and be like, “Oh snap. I forgot to do my chore.”...But then I’m right back on it.

SP403, unhoused

Several participants realized that they were using drugs and alcohol more than they thought by logging their substance use every 2 hours:

Oh my gosh, I was shocked how many times I actually put down how much I drank alcohol, and truthfully, I have been trying to stop.

SP408, unhoused

Furthermore, substance use was also linked to social and physical environments for some:

It also gave me a good understanding of how when I hang out with these certain people, yeah, I am smoking more. And if I hang out with certain people, I am drinking more...Gonna be like, okay dude, I’m hanging out with you but just because we’re hanging out doesn’t mean we have to drink. You know? If they’re drinking it’s their choice. Alright man, I’m noticing I can’t be hanging out with you every day. You’re drinking every day, I'm hanging out with you every day, I’m probably gonna be drinking every day.

SP406, unhoused

Questions about drug use and sexual activity were often considered sensitive topics—topics that others might not ask the participants about because of their sensitive nature. Some participants felt that the EMA prompts provided a safe and comfortable opportunity to check in:

I think you get to know more about yourself by...You’re being asked questions that a whole lot of people wouldn’t ask you. So I found it interesting for a device or a system, that’s not human, obviously, to ask you those questions. I was like: Wow. They were pretty insightful. Even if they were like the same old questions over and over and over. “How much drugs do you take?” Or “Who do you sleep with?”. It’s information that you want to keep private. You don’t want to share it with anybody. And also, it’s kind of a comfortable feeling too.

SP105, housed

In addition, the *check in* and ability to confide in the surveys seemed to provide a sense of calm for participants: such as who stated:

It took me a little break, too. That was my little break, with the alarm going off. I was like oh shit, I'm on my little couple seconds break.

SP403, unhoused

#### Self-reflection Negative Case Analysis: Causing Stress and Anxiety

Although self-reflection and self-awareness were common in focus group discussions, which seemed to promote a sense of calmness among participants, aspects of the EMA protocol also seemed to trigger stress, anxiety, and paranoia, particularly in response to location tracking. Approximately 16% (37/231) of participants reported EMA caused *somewhat* or *quite a bit* of stress or anxiety in the exit survey (Table 2). Although some participants liked the personal nature of the questions, allowing them to check in with themselves regarding personal behaviors that were otherwise likely to go unchecked, others felt that the questions were too personal. In fact, a participant discussed paranoia as a result of the prompts:

I got paranoid sometimes, like if I was hanging out with these people and it was asking me, “who are you with?” “Did you do any drugs with?”...It was just about the whole street thing; it feels like snitching.

SP301, unhoused

Similarly, another participant spoke of the discomfort they felt regarding the nature of the questions and location monitoring:

I did [feel uncomfortable] at first. I thought it was like the Feds or something. I was like oh shit, I’m not gonna lie, I was doing all types of lies...I felt like the Feds was watching. And taking video at the same time. I let the phone die for like a day and a half...I’m like I don’t know, I’m gonna keep the phone off. I was just annoyed about it them because like they know oh really alerted when I got a text that “we see your phone hasn't been charged.” [laughs] You guys are watching me! I know what you can do with technology. It’s a simple program, there’s no telling what’s written in that program that I don’t see.

SP403, unhoused

Fears regarding the personal nature of questions and location tracking, such as those discussed by participants SP301 and SP403, who were unhoused and both enrolled via drop-in centers, were more common among young adult participants who were actively homeless (ie, focus groups 3 and 4 [see Table 5 for more quotes by focus group and housing status]).

Further stress appeared to arise from the repetitive nature of the prompting schedule, with EMA prompts approximately 2 hours apart with the same array of questions. A participant commented on this by saying the following:

Well, it was irritating, like once or twice. I thought it was going to ask different questions. I didn’t know about the repetition thing.

SP106, housed

Some participants felt that the prompting occurred too frequently and reported that the prompt changed their mood and what they reported as the prompt itself annoyed them:

Sometimes it would annoy me, like, “How do you feel?” Like, oh, I feel annoyed now

SP207, housed

A similar response was recorded by another participant, who commented the following:

Damn, this alarm’s going off and now I’m irritated. Like I was doing fine before but now I’m irritated as fuck.

SP202, housed

An additional point about the prompting scheme focused on the concept of negativity in survey items, as a few participants noted that the surveys did not ask about positive things in their lives, such as work, school, or other productive aspects of their lives. A participant commented the following:

It seemed to always be focused on like, what did you do that wasn’t productive today. How much weed did you smoke how many did you smoke, how much did you drink, did you fuck anybody for drugs, what time did you wake up, did you even go to sleep tonight—it didn’t ask anything like I don’t know did you have a good day or did you...Did anything positive happen to you today. It was just all focused on, I’m not going to say negativity...I guess how often homeless youth use drugs and trade that for sex or whatever else. Being on the streets, just assuming being on the streets that’s all that you do.

SP403, unhoused

Despite these concerns, the participants seemed to become used to the prompting schedule over the course of the week. Specifically, a participant discussed their experience of becoming familiar with the surveys, which made the experience easier:

I got used to it...At first it was kind of slow, for me. So it’d take a while, maybe five or ten minutes. I’m taking forever on this thing until eventually it was two minutes. And after a while it wasn’t really bothering me.

SP404, unhoused

Considering the demands of the prompting schedule, participants had to juggle their study responsibilities with the responsibilities of their daily lives. Remaining stresses that resulted from study participation focused on having to comply (ie, answer a certain number of prompts) to get paid; needing to keep track of their study phones and answer surveys when at work or school; and not being comfortable with answering in front of others, such as SP408 when in the company of their case manager:

I know I didn’t want to miss one because you’re not gonna get your money. I want to get to a good percentage, high percentage

SP404, unhoused

If I was working or if I was at school...I want to get the survey done, but I really have to pay attention to what I’m doing. So it’s like let me just get it over with

SP103, housed

A few times it ringed off when I was in a meeting with my case manager. I was like “oh...no.” I couldn’t just pull out my phone and start doing a survey in front of my case manager. I was like, why you gotta hit at this hour.

SP408, unhoused

On occasion, these demands resulted in participants clicking through the surveys. A participant described this as “going into default”:

I did find myself going into a default and then just changing it if it was different because my mood was fairly stable throughout the day and I’d be like, “This one, this one, this one, this one.” Then I’m like, “Eh, this one’s actually over here now.” I definitely would go into a default but then I’d adjust it.

SP202, housed

#### Inciting Behavior Change

Approximately 22.9% (53/231) of the SPs thought that their behaviors had changed because of study participation. Most notably, behavior change was focused on drug and/or alcohol use and more often reported by unhoused participants than those residing in supportive housing. On some occasions, EMA promoted positive change, such as the intention to consume less of a substance, such as with one participant’s alcohol consumption:

I’m not going to drink today, because they’re going to ask me how many drinks I drank.

SP102, housed

However, on the other hand, prompting regarding drug or alcohol consumption also had negative impacts, resulting in cravings or the desire to consume. A participant explains the effect as follows:

I felt a little like after a day or two, I had kind of like a Pavlov’s dog effect, where every time it went off, I’m like, “Ooh, I could really use some alcohol right now.” I’m like, “Ooh, this is reminding me that alcohol is not a great option, but it is an option.”

SP206, housed

Similarly, another participant stated the following:

On the alcohol and drink question...the repetitive asking you another question and just mentioning a drink made me want to drink...It kept asking me how many drinks I have had and I’m like, none, but I don’t know, I kind of want one now, shit.

SP403, unhoused

In addition, the subject matter, combined with the annoyance of the prompting schedule, brought on an increased desire to use:

Especially since it was that ringing and a little bit of an irritant, and it’s like, “Ooh, a drink would be really nice to kind of just not deal with this right now”

SP206, housed

#### Responding Honestly

Across the 4 focus groups, the participants discussed honesty in their survey responses. Although 81.8% (189/231) of focus group participants agreed with being open and honest, others expressed greater distrust and, therefore, less honesty. Some participants oscillated between providing an honest picture of their day and, at times, lying. One of the participants provides a good example of the latter:

I should say...I was just trying to be as honest as possible. But some days you just don’t feel like explaining yourself or completely answering.

SP103, housed

Here, fluctuations in fatigue and social engagement influenced honesty in survey responses.

The occasions where participants reported dishonesty, which occurred more often among unhoused participants, were largely related to the personal nature of the questions. One of the participants noted that the more specific and personal the questions got, the harder they were to answer honestly. This participant continued by providing a specific example:

It was asking me to put a nickname down for someone you had sex with, I was like, I don’t really want to do that.

SP103, housed

Furthermore, another participant described their experience with honest survey responses as follows:

To be honest, I lied on a few questions...I lied because it just reminded me of what a whore I am. It would be like “How many times did you have sex today?” And stuff like that, I’m like it’s clocking me. I had the times I was cheating on my boyfriend with this boy, and you know how it asks you like the five people that you hang out with mostly or whatever? Then it would be like, “Who were you with?” I’d be like, “Ooh.”

SP202, housed

Despite these circumstances, many participants discussed why they chose to respond honestly to the survey items. A participant shared their respect for the study by stating the following:

I tried to be as honest as possible. Because I knew that it was a study. So I tried to be as honest...Because you guys are going to look at it and try to get real answers from people. It was hard. But I tried to be honest about it.

SP102, housed

Others noted that responding via the phone provided some comfort:

The thing about surveys too, is it’s a non-judgmental environment that you’re telling your information to.

SP105, housed

Another participant also had similar feelings about using their phone to answer the surveys honestly:

Yeah, I feel like if anything it makes me feel more comfortable, because the phone, you don’t have to give a fuck...I’m going to be real today, and I’m going to be real to myself. It’s kind of like a diary if you think about it. That survey was like a diary for a week.

SP205, housed

#### Suggestions for Future Studies: Technology

The largest perk for many participants in the study without a phone of their own was receiving a phone with a full data plan for the whole week, which most participants chose to do. Only 9.1% (21/231) of participants used their own phones for the study. Participants from focus group 4 (unhoused) discussed the following:

[Borrowing] the phone...actually helped me with my daily life, bro. Because I didn’t have a phone at the time, so it helped me to get phone calls.

SP404

Oh yeah, me too. Google maps.

SP408

I hadn’t had a phone since that one. I do want a phone for another week though.

SP404

Some others with their own personal devices preferred using their own phones rather than needing to keep track of 2 phones for the duration of the study:

I think [using my own phone] was more convenient. It was a lot easier than having to have a second phone. Losing track of it.

SP105, housed

I thought it was interesting. It was cool. I think I prefer it to be on my actual phone than another phone. Because it was kind of hard to keep up with it.

SP104, housed

In addition, some with their own personal phones chose to borrow a study phone to keep the study separate from their personal lives or because the software was incompatible with their phones. This, most often, resulted in difficulty keeping track of the 2 phones, as discussed in focus group 2 (housed):

It felt extra for me but that's because...I’m really bad about keeping my regular phone on me, so I said I was a bad millennial. I’m a bad millennial.

SP204

It’s hard to remember to carry two phones.

SP208

But even if I went from my bedroom to the dining room, and thirty minutes went by and I was like, “I have to go get my phone.” Then I saw that I missed one survey.

SP204

I feel like I have bigger problems than carrying two phones. It wasn’t the hardest thing in my life, but sometimes I would forget it and be like, “Aw, crap.” That's it.

SP207

Another discussion point stemming from this issue occurred in friend groups in which multiple people were enrolled in the study at the same time. One of the participants mentioned the difficulty of keeping track of their phone when interacting with others also in the study:

It was hard for me. I was literally driving. I always keep my phone away. I’m over there trying to jump in the purse like: Where is this phone? And then, another thing, my neighbor...My neighbor [who was also in the study] always comes over...And my other neighbor...So he’d be like: “Who’s phone is who’s? Who got that...” And I’d be like: “Do you have my phone? That's my phone?” How did we know it was our phone?...It took me about a day, or maybe two, to actually [get used to it].

SP104, housed

To this point, another participant in an unhoused focus group offered a potential way of distinguishing study phones in the same friend group, recommending the following:

Maybe have a choice of different ringtones, because when you’re in a group of people and the ringtone goes off, but everyone thinks it’s theirs...Everyone’s like, “Hold on, I think it’s mine.”

Finally, and perhaps most notably, SPs recognized the value of EMA as an intervention. Participants wanted more, such as prompts to support them in cutting back on their substance use, with many participants noting the benefits of study participation in terms of mindfulness:

I feel like sometimes you’re doing stuff and then...Like, say somebody says yes, to something on the list. Like some serious drugs...Meth or something like that. Which you know is a serious drug. You can tell them like: “Maybe you shouldn't do it.” Or something like that. I’m not sure about the whole thing. I’d have to sit down and go over it myself. If I was designing the app or something.

SP101, housed

### Mixed Methods

In comparing the quantitative and qualitative arms of this study via a convergent parallel design, we found qualitative responses from both housed and unhoused participants to confirm the quantitative findings (see Table 6 for an integration of the quantitative and qualitative findings). The findings regarding the previously discussed difficulties with charging devices were found to be convergent. Qualitative findings regarding study-induced stress and anxiety, as well as interference with daily life, provided additional contextual information to the quantitative findings, offering an expansion in the interpretation of results. As previously stated, unhoused participants self-reported greater, statistically significant study-induced stress and anxiety. However, the qualitative findings highlight that housed individuals, at times, also noted stress and anxiety related to study participation; however, this stress and anxiety seemed to be contextually different from that experienced by unhoused participants, which was often connected to paranoia, fears of snitching, and being *watched by the Feds*. The latter stress and anxiety could be directly related to street culture and economy. Similarly, quantitative findings showed increased reporting of study interference in daily life among unhoused individuals; however, qualitative findings showed that both unhoused and housed participants noted interference. Housed participants most often talked about interference in terms of school or work responsibilities, whereas those unhoused discussed needing to answer to make the money for survey compliance, which made it more likely to interfere with what they were doing.

**Table 6:** Results of convergent parallel design based on housing status.

Quantitative findings	Qualitative findings	Merged findings outcome
Compared with housed participants, unhoused participants were more likely to report that the *study caused them stress or anxiety* (*P*=.02).	Although both housed and unhoused participants reported stress accompanying the study, unhoused participants discussed stress related to paranoia and snitching.	Confirmatory; expansion
The study was reported to have *interfered with daily life* among those unhoused compared with those in housing (*P*<.001).	Although surveys appeared at inopportune times for both housed (eg, while at work or school) and unhoused (eg, while visiting with a case manager) participants, unhoused participants discussed more stress regarding needing to answer to make the money for survey compliance, which made it more likely to interfere with what they were doing.	Confirmatory; expansion
Unhouse participants reported greater *difficulty charging* their devices (*P*=.02).	Housed participants were more likely to be in locations with outlets. Charging had to be sought out by unhoused participants.	Confirmatory; convergent
More unhoused participants reported that the study caused *changes in their behavior* (*P*<.001) than those in housing.	Although both housed and unhoused participants noted increased awareness of substance use related to substance use questions, unhoused participants more often reported increased substance use, whereas housed participants seemed to mention the awareness of substance use and trended toward a reduction in their use.	Confirmatory; complementarity
Compared with those in housing, unhoused participants reported being less likely to *be open or honest* when answering survey items (*P*=.03).	Housed participants seemed to feel more comfortable with honest responses, whereas unhoused participants again noted paranoia and fear of snitching.	Confirmatory; convergent

Additional statistically significant differences between housed and unhoused participants occurred regarding reported behavior changes and honesty in the survey responses. Although both housed and unhoused participants noted increased awareness of substance use related to substance use questions, quantitative findings showed that more unhoused participants reported that the study affected their behavior during the week. Qualitatively, we obtained complementary findings in that those who were unhoused more often reported increased substance use, whereas housed participants seemed to mention the awareness of substance use and trended toward a reduction in their use. Finally, convergent findings emerged regarding honesty in the responses. It was clear that housed participants seemed to feel more comfortable answering openly and honestly, perhaps as a result of the newfound freedom associated with transitioning from homelessness to housing [34]. This is contrasted by unhoused participants once again noting paranoia and fear of outing peers on the street.

## Discussion

### Principal Findings

The results of this mixed methods study illuminate the experiences of housed and unhoused young adults enrolled in an EMA for a 1-week period. Although no statistically significant differences in compliance by housing status were found, statistically significant differences were found regarding the impact of study participation. Housing status was found to affect young adults’ engagement with EMA. Differences in housing status were found regarding the ability to keep their device charged, interference with daily life, stress and anxiety associated with participation, behavior change as a result of EMA, and the ability to respond openly and honestly to prompts.

In terms of compliance, those who had difficulty charging also had lower survey compliance when the phone was charged, and unhoused participants reported greater difficulty in charging. Perhaps the unhoused participants had difficulty charging but made sure to find some way to charge it, possibly because of the importance of the incentive. Similarly, less interference with daily life was associated with greater study compliance in both the daily and EMA surveys, and unhoused individuals noted greater interference. A thought here is the significant correlation between difficulty in charging the device and interference in daily life. Research apps may drain battery life more quickly than other apps and disrupt typical charging patterns based on their usual phone use. If unhoused participants struggled to find a power source and spent much time consumed with finding ways to charge the phone [35] to maintain compliance (ie, get paid), this could interfere greatly with one’s day. This could also explain why unhoused participants reported greater stress and anxiety associated with study participation. Again, status was not associated with compliance rates; however, greater stress and anxiety resulting from study participation produced worse compliance rates.

Overall, study compliance was approximately 80%, with no significant detected differences explicitly related to housing status. However, this does not imply that housing status does not need to be considered in the study design. The findings clearly reveal greater impacts, and perhaps burden, for unhoused participants, which was confirmed in the qualitative interviews. Future use of EMA, particularly with unhoused individuals, should consider barriers to using technology in research. Although entirely possible, a study design that relies on the use of technology such as a mobile device should consider issues related to access and how this may create increased burdensomeness. We observed how increased burden could produce additional stress in an already stressful environment, particularly with regard to being tracked. In some cases, the awareness of being tracked has the potential to exacerbate the underlying mental health or substance use issues. Greater efforts to ensure comfort with protocols, including an enhanced focus on confidentiality, could be beneficial, especially because of the histories of marginalization that have led to a deep-seated distrust of systems, including social service systems. Taking more time at the outset of EMA studies to ensure understanding and consent could promote greater trust and decrease the associated stress and anxiety. This could also increase honesty in responses, particularly among unhoused individuals who reported being less honest in their responses compared with those in supportive housing, potentially because of perceived impacts on securing housing or other needed services and resources. Other methods to potentially increase data quality and assurance are to use a lead-in period where participants would get a day or two of practice with the app and EMA questions before recording their responses for analysis.

In testing for reactivity using anxiety and depression symptomatology before and after EMA the week, results show compliance to not be significantly related to the degree of anxiety and depression symptomatology reported. However, a decrease in reported symptoms occurred from before to after the test. This is particularly interesting as some participants, particularly unhoused participants, discussed increased stress and anxiety because of study participation. This indicates that the momentary stress of being prompted did not affect symptomatology and perhaps was fleeting. Although participants may have frequently thought about the bother of the study and became momentarily overwhelmed, it seems to have not been a lasting experience with long-term impacts, despite 22.9% (53/231) of participants feeling that the study affected their behaviors. The fact that compliance was not significantly related to symptomatology (ie, more prompts completed were associated with increases in symptomatology) indicates low reactivity to EMA prompting and participation. More work is needed to effectively examine the overall decreases in symptomatology that may be associated with EMA, as the results cannot definitively be ruled as reactivity, echoing previous literature [36].

The study’s implications include support for using intensive longitudinal methods such as EMA with housed and unhoused young adults. Acorda et al [26] explored the impact and acceptability of EMA among 18 youths experiencing homelessness, making recommendations for use with young people who are actively homeless. The results produced by Acorda et al [26] reinforce the findings of this study, most notably the effects of increased self-awareness and the potential for behavior change as a result of EMA. Given the discussion regarding perceived behavioral change and behavioral intentions, EMA also presents possible opportunities for intervention work. It is clear from both their work and the additional support offered from these analyses that EMA is highly acceptable for young adults who have experienced homelessness, both housed and unhoused, with several special considerations, particularly around housing status and confidentiality.

### Limitations

Despite the strengths of analyzing this innovative method for vulnerable young adults, there are several limitations that must be acknowledged. The results suggest that some participants did not always answer honestly. We do not have a way of knowing the responses to trust or not using this method with this population. Future work may want to design studies to test the validity of this method with this population, perhaps adapting the study design to include *self-checks* within survey protocols and questions about honesty at the conclusion of each EMA survey. In addition, relying on focus groups as the qualitative methodology could potentially lead to group think more than individual interviews, particularly regarding sensitive topics where group interactions could be detrimental to the discussion. Inquiring about sensitive topics is needed for epidemiological studies; however, a focus solely on what may be perceived as *negative* aspects without the inclusion of a strengths-based perspective lacks an equity lens and was felt by some SPs. Finally, we considered the difference between completion and compliance within the EMA context and ultimately decided to use compliance as the number of prompts completed out of those received based on the study aims. Care should be taken when interpreting findings, specifically if the interest is in distilling information regarding the technological aspects of EMA methods, as this study did not consider technical issues as a focus of analysis, although it is briefly reported.

### Conclusions

Although with caveats, this study produced evidence in favor of the use of intensive longitudinal designs with young adults who were formerly and are currently homeless. Intensive longitudinal methods are well suited to capture experiences associated with the chaotic and unstable environments of homelessness, addressing the limitations of cross-sectional and traditional longitudinal designs. However, the findings of this study show that chaotic and unstable environments must be considered at every step of the research process. These findings have implications for research development and design, data collection, and analysis.

## Appendix

Multimedia Appendix 1 Ecological momentary assessment questionnaire.

Multimedia Appendix 2 Daily survey questions.

Multimedia Appendix 3 Focus group guiding questions.

## Abbreviations

EMA: ecological momentary assessment
SP: study participant
