# Supplementary material for: Feasibility and Acceptability of Ecological Momentary Assessment With Young Adults Who Are Currently or Were Formerly Homeless: Mixed Methods Study
Source: JMIR Form Res. 2022 Mar 25;6(3):e33387. doi: 10.2196/33387 (PMC8994151; doi:10.2196/33387)
Supplement: Multimedia Appendix 1 [file formative_v6i3e33387_app1.pdf]

Appendix 1. EMA Questionnaire

Q1_social. Over the **past 2 hours**, who have you interacted with in any way?

- ☐ [Person 1, entered at baseline setup]
- ☐ [Person 2, entered at baseline setup]
- ☐ [Person 3, entered at baseline setup]
- ☐ [Person 4, entered at baseline setup]
- ☐ [Person 5, entered at baseline setup]
- ☐ Someone else not listed here
- ☐ I have not interacted with anyone

[Display if "Someone else not listed here" is selected for Q1_socialcore.]

Q1_a_socialother. Over the **past 2 hours**, who else have you interacted with? (check all that apply)

- ☐ Friends from home/before you were homeless
- ☐ Friends or peers from the street or an agency
- ☐ Family (biological or foster)
- ☐ Romantic/sexual partner
- ☐ Case worker or agency staff/volunteer
- ☐ People from work or school
- ☐ Law enforcement (police, security, etc.)
- ☐ Someone I don't know well/random person

Q2_where. Where are you **currently**?

- ☐ My apartment/residence
- ☐ Someone else's residence
- ☐ In transit (bus, car, etc.)
- ☐ Outdoors (park, beach, sidewalk, etc.)
- ☐ School or work
- ☐ Social service agency (drop-in, shelter, DPSS, etc.)
- ☐ Place of business (restaurant, bar, mall, etc.)
- ☐ Other

Q2_safe. Just **before the phone went off**, how **SAFE** did you feel?

- ○ Very unsafe
- ○ Somewhat unsafe
- ○ Neither safe nor unsafe
- ○ Somewhat safe
- ○ Very safe

Q2_what. Just **before the phone went off**, what was the main thing you were doing?

- ○ Going somewhere
- ○ Hanging out
- ○ Working/job/school
- ○ Meal/eating food
- ○ Sleeping/resting
- ○ Appointment/meeting
- ○ Other

Q3_happy. Just **before the phone went off**, how **HAPPY** were you feeling?

- ○ Slightly/not at all

- ○ A little
- ○ Moderately
- ○ Quite a bit
- ○ Extremely

Q4_stressed. Just **before the phone went off**, how **STRESSED** were you feeling?
- ○ Slightly/not at all
- ○ A little
- ○ Moderately
- ○ Quite a bit
- ○ Extremely

Q5_sad. Just **before the phone went off**, how **SAD / DEPRESSED** were you feeling?
- ○ Slightly/not at all
- ○ A little
- ○ Moderately
- ○ Quite a bit
- ○ Extremely

Q6_irritated. Just **before the phone went off**, how **IRRITATED** were you feeling?
- ○ Slightly/not at all
- ○ A little
- ○ Moderately
- ○ Quite a bit
- ○ Extremely

Q7_calm. Just **before the phone went off**, how **CALM/RELAXED** were you feeling?
- ○ Slightly/not at all
- ○ A little
- ○ Moderately
- ○ Quite a bit
- ○ Extremely

Q8_excited. Just **before the phone went off**, how **EXCITED** were you feeling?
- ○ Slightly/not at all
- ○ A little
- ○ Moderately
- ○ Quite a bit
- ○ Extremely

Q9_bored. Just **before the phone went off**, how **BORED** were you feeling?
- ○ Slightly/not at all
- ○ A little
- ○ Moderately
- ○ Quite a bit
- ○ Extremely

Q10_hungry. Just **before the phone went off**, how **HUNGRY** were you feeling?
- ○ Slightly/not at all
- ○ A little
- ○ Moderately

- ○ Quite a bit
- ○ Extremely

Q10_stressevents. Over the **past 2 hours**, did any of these things happen to you? (check all that apply)

- ☐ I felt threatened or harassed
- ☐ Verbal fight or argument
- ☐ Physical fight
- ☐ I got injured or became ill
- ☐ Received bad news about something important
- ☐ Received good news about something important
- ☐ Interaction with security/law enforcement
- ☐ None of the above

Q11_tobacco. Over the **past 2 hours**, have you used… (check all that apply)

- ☐ I have not used tobacco
- ☐ Paper cigarettes
- ☐ E-cigarettes/vaped tobacco
- ☐ Chewing tobacco/dip
- ☐ A tobacco product not listed here

Q12_alcohol. Over the **past 2 hours**, how many <u>alcoholic drinks</u> did you have?

- ○ 0
- ○ 1
- ○ 2
- ○ 3
- ○ 4
- ○ 5 or more

[Display if NOT "0" to Q12_alc.]

Q12_b_alcohol_where. Where were you **when you used alcohol**? (check all that apply)

- ☐ My apartment/residence
- ☐ Residence of someone else
- ☐ In transit (bus, car, etc.)
- ☐ Outdoors (park, beach, sidewalk, etc.)
- ☐ School or work
- ☐ Social service agency (drop-in, shelter, DPSS, etc.)
- ☐ Place of business (restaurant, bar, mall, etc.)
- ☐ Other

[Display if NOT "0" to Q12_alc.]

Q12_c_alcohol_who. Who were you with **when you used alcohol**? (check all that apply)

- ☐ [Person 1, entered at baseline setup]
- ☐ [Person 2, entered at baseline setup]
- ☐ [Person 3, entered at baseline setup]
- ☐ [Person 4, entered at baseline setup]
- ☐ [Person 5, entered at baseline setup]
- ☐ Someone else not listed here
- ☐ Nobody

[Display if "Someone else not listed here" is selected for Q12b_alc_whocore.]

Q12_d_alcohol_who_other. Who else were you with **when you used alcohol**? (check all that apply)

- ☐ Friends from home/before you were homeless
- ☐ Friends or peers from the street or an agency
- ☐ Family (biological or foster)
- ☐ Romantic/sexual partner
- ☐ People from work or school
- ☐ Someone I don't know well/random person

[Display if NOT "Nobody" to Q12b_alc_whocore.]
Q12_e_alcohol_who_use. Were any of the people with you using alcohol?
- ○ Yes
- ○ No
- ○ Not sure

Q13_drugs. Over the **past 2 hours**, have you used <u>any drugs</u>?
- ○ Yes
- ○ No

[Display if "Yes" to Q13_drug.]
Q13_a_drugs_type. What substances did you use over the **past 2 hours**? (check all that apply)
- ☐ Marijuana
- ☐ Meth
- ☐ Ecstasy / MDMA / "Molly"
- ☐ Synthetic marijuana (K2, Spice, etc.)
- ☐ Hallucinogens/psychedelics
- ☐ Prescription drugs, not as prescribed (Rx cough syrup, Oxycontin, Xanax, etc.)
- ☐ Heroin
- ☐ Other

[Display if "Other" is selected in Q14a_drug_type]
Q13_a8_drugs_type_other. What other drug did you use? (please specify)
________________________________ (text entry)

[Display if "Yes" to Q14_drug.]
Q13_b_drugs_where. Where were you **when you used drugs**? (check all that apply)
- ☐ My apartment/residence
- ☐ Someone else's residence
- ☐ In transit (bus, car, etc.)
- ☐ Outdoors (park, beach, sidewalk, etc.)
- ☐ School or work
- ☐ Social service agency (drop-in, shelter, DPSS, etc.)
- ☐ Place of business (restaurant, bar, mall, etc.)
- ☐ Other

[Display if "Yes" to Q14_drug.]
Q13_c_drugs_who. Who were you with **when you used drugs**? (check all that apply)
- ☐ [Person 1, entered at baseline setup]
- ☐ [Person 2, entered at baseline setup]
- ☐ [Person 3, entered at baseline setup]
- ☐ [Person 4, entered at baseline setup]
- ☐ [Person 5, entered at baseline setup]
- ☐ Someone else not listed here

- ☐ Nobody

[Display if “Someone else not listed here” is selected for Q14c_drug_whocore.]
Q13_d_drugs_who_other. Who else were you with **when you used drugs**? (check all that apply)
- ☐ Friends from home/before you were homeless
- ☐ Friends or peers from the street or an agency
- ☐ Family (biological or foster)
- ☐ Romantic/sexual partner
- ☐ People from work or school
- ☐ Someone I don’t know well/random person

[Display if NOT “Nobody” to Q14c_drug_whocore.]
Q13_e_drugs_who_use. Were any of the people you were with using drugs?
- ○ Yes
- ○ No
- ○ Not sure

[Only display the remaining questions if “0” to Q13_alc. AND “No” to Q14_drug.]
Q14_tempted. Over the **past 2 hours**, did you think about using drugs or alcohol?
- ○ Yes
- ○ No

[Display if “Yes” to Q15_tempt.]
Q14_a_tempted_where. Where were you **when you thought about using** drugs or alcohol? (check all that apply)
- ☐ My apartment/residence
- ☐ Someone else’s residence
- ☐ In transit (bus, car, etc.)
- ☐ Outdoors (park, beach, sidewalk, etc.)
- ☐ School or work
- ☐ Social service agency (drop-in, shelter, DPSS, etc.)
- ☐ Place of business (restaurant, bar, mall, etc.)
- ☐ Other

[Display if “Yes” to Q15_tempt.]
Q14_b_tempted_who. Who were you with **when you thought about using** drugs or alcohol? (check all that apply)
- ☐ [Person 1, entered at baseline setup]
- ☐ [Person 2, entered at baseline setup]
- ☐ [Person 3, entered at baseline setup]
- ☐ [Person 4, entered at baseline setup]
- ☐ [Person 5, entered at baseline setup]
- ☐ Someone else not listed here
- ☐ Nobody

[Display if “Someone else not listed here” is selected for Q15b_tempt_whocore.]
Q14_c_tempted_who_other. Who else were you with **when you thought about using** drugs or alcohol? (check all that apply)
- ☐ Friends from home/before you were homeless
- ☐ Friends or peers from the street or an agency
- ☐ Family (biological or foster)

- ☐ Romantic/sexual partner
- ☐ People from work or school
- ☐ Someone I don’t know well/random person

[Display if NOT “Nobody” to Q15b_tempt_whocore.]
Q14_d_tempted_who_use. When you thought about using drugs or alcohol, were any of the people you were with <u>using drugs or alcohol</u>?
- ○ Yes
- ○ No
- ○ Not sure

Q15_thankyou. [thank you end of survey screen] ###END OF SURVEY###
