# Supplementary material for: Feasibility and Acceptability of Ecological Momentary Assessment With Young Adults Who Are Currently or Were Formerly Homeless: Mixed Methods Study
Source: JMIR Form Res. 2022 Mar 25;6(3):e33387. doi: 10.2196/33387 (PMC8994151; doi:10.2196/33387)
Supplement: Multimedia Appendix 2 [file formative_v6i3e33387_app2.pdf]

Appendix 2. Daily survey questions

Part I: Sleep Questions

Q1_waket. What time did you wake-up **today**?
[time picker, now in 15 min intervals on a scroll wheel instead of Android default time picker] + "I did not sleep last night" box
[If "I did not sleep last night" is selected, Skip to Part II Substance Use Questions]

Q2_sleept. What time did you go to sleep **yesterday**?
[time picker, now in 15 min intervals on a scroll wheel instead of Android default time picker]

Q3_sleeploc. Where did you sleep **last night**?

- ○ My own apartment/residence
- ○ Home of someone I know (family, friend, partner, etc.)
- ○ Stranger's home/residence
- ○ Shelter/transitional living program
- ○ Hotel/motel
- ○ Abandoned building, squat, public transit, or other place inside
- ○ Street, park, beach, roof, or other place outside
- ○ Car, RV, van, or other vehicle
- ○ Other, please specify: __________

[Display if "Other" to Q3_sleeploc.]
Q3_sleeploc_other. What other place did you sleep **last night**?
__________________ (text entry)

Q4_sleepq. Last night, my sleep quality was:

- ○ Very poor
- ○ Poor
- ○ Fair
- ○ Good
- ○ Very good

Part II: Substance Use Questions

Q5_soccore. At any point **yesterday**, did you interact with any of these people in any way? (check all that apply)

- ☐ [Person 1, entered at baseline setup]
- ☐ [Person 2, entered at baseline setup]
- ☐ [Person 3, entered at baseline setup]
- ☐ [Person 4, entered at baseline setup]
- ☐ [Person 5, entered at baseline setup]
- ☐ I did not interact with any of these people **yesterday**

Q6a_subchk1. At any point **yesterday**, did you use any of these substances? (check all that apply)

- ☐ Alcohol
- ☐ Marijuana
- ☐ Synthetic marijuana (K2, spice, etc.)
- ☐ Meth
- ☐ Prescription drugs, not as prescribed (Rx cough syrup, Oxycontin, Xanax, etc.)
- ☐ Other illicit drug
- ☐ I did not use any drugs yesterday

[Display if "Other illicit drug" was selected in Q6a_subchk1.]

Q6_6_subchk2. What other illicit drug(s) did you use **yesterday**? (check all that apply)

- ☐ Ecstasy / MDMA / "Molly"
- ☐ Hallucinogens/psychedelics
- ☐ Heroin
- ☐ Cocaine or crack
- ☐ Something else not listed here

[Display if "something else not listed here" (17) was selected in Q6b_subchk2]:

Q6_11_other. You indicated that you used something else that wasn't listed. How did you use that/those other drug(s) **yesterday**? (check all that apply)

- ☐ Swallowed
- ☐ Smoked
- ☐ Snorted
- ☐ Injected
- ☐ Other

[Display if "alcohol" was selected in Q6a_subchk1.]

Q6_01_alc. How many <u>alcoholic drinks</u> did you have **yesterday**?

[number picker; range=0-99]

Note: "One drink" is:

- a 12-oz beer
- 1/5 of a 40-oz bottle of malt liquor
- a 5-oz glass of wine
- a 1.5 oz "shot" of 80-proof liquor

[Display if response to Q6_01_alc. is ≥ 4/5 (4 for women/5 for men)]

Q6_01a_alcbinge. At any point **yesterday**, did you drink <u>4/5</u> [4 for women/5 for men] <u>or more drinks</u> **in less than a 2-hour period?**

- ○ Yes
- ○ No
- ○ Not sure

[Display if "alcohol" was selected in Q6a_subchk1.]
Q6_01b_alcwhocore. At any point **yesterday**, did you drink alcohol with any of these people? (check all that apply)
- ☐ [Person 1, entered at baseline setup]
- ☐ [Person 2, entered at baseline setup]
- ☐ [Person 3, entered at baseline setup]
- ☐ [Person 4, entered at baseline setup]
- ☐ [Person 5, entered at baseline setup]

[Display if "marijuana" was selected in Q6a_subchk1.]
Q6_02a_marj. How many times did you use marijuana or marijuana products **yesterday?**
[number picker; range=0-99]

Q6_02b_marj. At any point **yesterday**, did you use marijuana concentrates? (hashish, hash oil, "dabs," etc.)
- ○ Yes
- ○ No
- ○ Not sure

Q6_02c_marjwhocore. At any point **yesterday**, did you use marijuana or marijuana products with any of these people? (check all that apply)
- ☐ [Person 1, entered at baseline setup]
- ☐ [Person 2, entered at baseline setup]
- ☐ [Person 3, entered at baseline setup]
- ☐ [Person 4, entered at baseline setup]
- ☐ [Person 5, entered at baseline setup]
- ☐ I did not use marijuana or marijuana products with any of these people **yesterday**

[Display if "synthetic marijuana" was selected in Q6a_subchk1.]
Q6_03a_synmj. How many times did you use synthetic marijuana **yesterday**? (K2, spice, fake weed, etc.)
[number picker; range=0-99]

Q6_03b_synmjwhocore. At any point **yesterday**, did you use synthetic marijuana with any of these people? (check all that apply)
- ☐ [Person 1, entered at baseline setup]
- ☐ [Person 2, entered at baseline setup]
- ☐ [Person 3, entered at baseline setup]
- ☐ [Person 4, entered at baseline setup]
- ☐ [Person 5, entered at baseline setup]
- ☐ I did not use synthetic marijuana with any of these people **yesterday**

[Display if "meth" was selected in Q6a_subchk1.]
Q6_04a_meth. How many times did you use meth **yesterday**?
[number picker; range=0-99]

Q6_04b_meth. How did you use meth **yesterday**? (check all that apply)
- ☐ Smoked

- ☐ Snorted
- ☐ Injected
- ☐ Swallowed

Q6_04c_methwhocore. At any point **yesterday**, did you use meth with any of these people? (check all that apply)

- ☐ [Person 1, entered at baseline setup]
- ☐ [Person 2, entered at baseline setup]
- ☐ [Person 3, entered at baseline setup]
- ☐ [Person 4, entered at baseline setup]
- ☐ [Person 5, entered at baseline setup]
- ☐ I did not use meth with any of these people **yesterday**

[Display if "prescription drugs…not prescribed…" was selected in Q6a_subchk1.]
Q6_05a_pdm. How many times did you use prescription drugs that were not prescribed, or in higher doses than prescribed, **yesterday**?

[number picker; range=0-99]

Q6_05b_pdm. How did you use prescription drugs, not as prescribed **yesterday**? (check all that apply)

- ☐ Swallowed
- ☐ Snorted
- ☐ Injected
- ☐ Smoked

Q6_05c_pdmwhocore. At any point **yesterday**, did you use prescription drugs, not as prescribed with any of these people? (check all that apply)

- ☐ [Person 1, entered at baseline setup]
- ☐ [Person 2, entered at baseline setup]
- ☐ [Person 3, entered at baseline setup]
- ☐ [Person 4, entered at baseline setup]
- ☐ [Person 5, entered at baseline setup]
- ☐ I did not use prescription drugs, not as prescribed with any of these people **yesterday**

[Display if "Ecstacy/MDMA/'Molly'" was selected in Q6a_subchk1.]
Q6_07a_mdma. How many times did you use Ecstacy/MDMA/"Molly" **yesterday**?

[number picker, range=0-99]

Q6_07b_mdma. How did you use Ecstacy/MDMA/"Molly" **yesterday**? (check all that apply)

- ☐ Swallowed
- ☐ Snorted
- ☐ Injected

Q6_07c_mdmawhocore. At any point **yesterday**, did you use Ecstacy/MDMA/"Molly" with any of these people? (check all that apply)

- ☐ [Person 1, entered at baseline setup]
- ☐ [Person 2, entered at baseline setup]
- ☐ [Person 3, entered at baseline setup]
- ☐ [Person 4, entered at baseline setup]
- ☐ [Person 5, entered at baseline setup]
- ☐ I did not use Ecstacy/MDMA/"Molly" with any of these people **yesterday**

[Display if “hallucinogens/psychedelics” was selected in Q6a_subchk1.]
Q6_08a_halluc. How many times did you use hallucinogens/psychedelics (LSD/acid, “shrooms,” etc) **yesterday?**
[number picker; range=0-99]

Q6_08b_hallucwhocore. At any point **yesterday**, did you use hallucinogens/psychedelics (LSD/acid, “shrooms,” etc) with any of these people? (check all that apply)
- ☐ [Person 1, entered at baseline setup]
- ☐ [Person 2, entered at baseline setup]
- ☐ [Person 3, entered at baseline setup]
- ☐ [Person 4, entered at baseline setup]
- ☐ [Person 5, entered at baseline setup]
- ☐ I did not use hallucinogens/psychedelics with any of these people **yesterday**

[Display if “heroin” was selected in Q6_6_subchk2.]
Q6_09a_heroin. How many times did you use heroin **yesterday**?
[number picker; range=0-99]

Q6_09b_heroin. How did you use heroin **yesterday**? (check all that apply)
- ☐ Snorted
- ☐ Injected
- ☐ Smoked

Q6_09c_heroinwhocore. At any point **yesterday**, did you use heroin with any of these people? (check all that apply)
- ☐ [Person 1, entered at baseline setup]
- ☐ [Person 2, entered at baseline setup]
- ☐ [Person 3, entered at baseline setup]
- ☐ [Person 4, entered at baseline setup]
- ☐ [Person 5, entered at baseline setup]
- ☐ I did not use heroin with any of these people **yesterday**

[Display if “cocaine or crack” was selected in Q6_6_subchk2.]
Q6_10a_coccrk. You indicated that you used cocaine or crack **yesterday**. Which type(s) did you use?
- ○ Powdered cocaine
- ○ Crack or freebase cocaine
- ○ Both

Q6_10b_coccrk. How many times did you use cocaine or crack **yesterday**?
[number picker; range=0-99]

Q6_10c_coccrk. How did you use cocaine or crack **yesterday**? (check all that apply)
- ☐ Orally (swallowed, chewed, rubbed on gums, etc.)
- ☐ Smoked
- ☐ Snorted
- ☐ Injected

Q6_10d_coccrk. At any point **yesterday**, did you use cocaine or crack with any of these people? (check all that apply)
- ☐ [Person 1, entered at baseline setup]
- ☐ [Person 2, entered at baseline setup]

- ☐ [Person 3, entered at baseline setup]
- ☐ [Person 4, entered at baseline setup]
- ☐ [Person 5, entered at baseline setup]
- ☐ I did not use cocaine or crack with any of these people **yesterday**

Part III: Sexual Activity Questions

Q7_partners. How many people did you have vaginal or anal sex with **yesterday**?
- 0
- 1
- 2
- 3
- 4 or more

[Loop the following questions for each partner (e.g., for two partners you'd have Q7_1_..., Q7_2_..., etc.)

Q7_1a_id. Please provide initials or a nickname for the **first** person you had vaginal or anal sex with **yesterday**:
________ (text input)

Q7_1b1_parttype. How would you describe [**PARTNER 1 IDENTIFIER**]**?**
- Serious partner (husband, wife, life partner, girlfriend, boyfriend, etc.)
- Casual partner (hookup, friends with benefits, one night stand, etc.)

Q7_1b2_partdur. How long have you been engaged in a sexual relationship with **[PARTNER 1 IDENTIFIER]**? (in total)
- 1 day or less
- 1-30 days
- 1-6 months
- 6-12 months
- 1-3 years
- More than 3 years

Q7_1c_partgndr. What is **[PARTNER 1 IDENTIFIER]'s gender identity?**
- Male
- Female
- Trans male/Trans man
- Trans female/Trans woman
- Genderqueer/Gender non-conforming
- Different identity (please specify _______________)

[Do not display if both respondent and partner are women]
Q7_1d_avnocndm. At any point **while having sex with [PARTNER 1 IDENTIFIER],** did you have vaginal or anal sex without a condom?
- No
- Yes, both anal and vaginal sex without a condom
- Yes, vaginal without a condom only
- Yes, anal without a condom only

[Do not display if NO to Q7_1d_avnocndm:]
Q7_1d1_HIV. Did you know **[PARTNER 1 IDENTIFIER]'s** HIV status before having vaginal or anal sex without a condom with them?
- Yes
- No

Q7_1e_SUI. Did you drink alcohol or use drugs before you had sex (vaginal or anal sex) with **[PARTNER 1 IDENTIFIER]?**

- ○ Yes
- ○ No

[Display for each partner in the Q7 loop:]

Q7_1f_sexwhere. Where were you when you engaged in sex with **[PARTNER 1 IDENTIFIER]**? (check all that apply)

- ☐ My home/residence
- ☐ The home of someone I know
- ☐ The home of someone I don't know or barely know
- ☐ Hotel/motel
- ☐ Outside (park, beach)
- ☐ Tent or improvised shelter
- ☐ Car, RV, or other vehicle
- ☐ Abandoned building or squat
- ☐ Shelter or drop-in
- ☐ Other (please specify): ___________________

Q7_2a_id. Please provide initials or a nickname for the **second** person you had vaginal or anal sex with **yesterday**:

___________(text input)

**[loop same follow-ups for each sex partner, w/ # of loops indicated by Q4_partners.]

[Always display:]

Q8_exchsex. At any point **yesterday**, did you trade any type of sex (oral, vaginal, or anal) for: (check all that apply)

- ☐ I did not trade sex
- ☐ Money
- ☐ Drugs
- ☐ A place to stay
- ☐ Food or meals
- ☐ Something else

[Display if NOT "I did not trade sex" to Q8_exchsex:]

Q8_1_exchsex_where. Where were you when you traded sex for money, drugs, a place to stay, food or meals, or something else? (check all that apply)

- ☐ My home/residence
- ☐ The home of someone I know
- ☐ The home of someone I don't know or barely know
- ☐ Hotel/motel
- ☐ Outside (park, beach)
- ☐ Tent or improvised shelter
- ☐ Car, RV, or other vehicle
- ☐ Abandoned building or squat
- ☐ Shelter or drop-in
- ☐ Other (please specify): ___________________
