# Supplementary material for: Feasibility and Acceptability of Ecological Momentary Assessment With Young Adults Who Are Currently or Were Formerly Homeless: Mixed Methods Study
Source: JMIR Form Res. 2022 Mar 25;6(3):e33387. doi: 10.2196/33387 (PMC8994151; doi:10.2196/33387)
Supplement: Multimedia Appendix 3 [file formative_v6i3e33387_app3.pdf]

Appendix 3. Focus group guiding questions

1. In general, how was your experience with the study? What did you like? What didn't you like?
   a. Did your participation in the study interfere with your daily life? If so, how much?
   b. Did responding to the Experience Surveys and Daily Logs throughout the week affect or influence your typical behavior? If so, how?
   c. Did you feel comfortable answering Experience Surveys and Daily Logs completely honestly and accurately? Why or why not? Ever misreport? Answer inaccurately?
      i. Comfortable answering all questions? Substance use? Sex? Who was with?
      ii. What might have made you feel more comfortable answering openly and honestly?
2. Do you have any suggestions for what you liked/disliked about the app or how the app developer could improve it?
      i. More or less surveys? Specific questions add or delete? Support?
3. Is there anything else you'd like to tell us about what you liked or disliked about the study or how it could be improved?
